# Genetics Investigation of Idiopathic Premature Ovarian Insufficiency: Contribution of Array-CGH and Next-Generation Sequencing

**DOI:** 10.3390/genes16030251

**Published:** 2025-02-22

**Authors:** Claire Cozette, Mathilde Pujalte, Noémie Celton, Dorian Bosquet, Henri Copin, Rosalie Cabry, Loic Garçon, Moncef Benkhalifa, Florence Scheffler, Guillaume Jedraszak

**Affiliations:** 1 Reproductive Medicine and Biology Department, CECOS of Picardy, Amiens University Hospital, 80000 Amiens, France; cozette.claire@chu-amiens.fr (C.C.); bosquet.dorian@chu-amiens.fr (D.B.); copin.henri@chu-amiens.fr (H.C.); cabry.rosalie@chu-amiens.fr (R.C.); benkhalifamoncef78@gmail.com (M.B.); 2 Department of Constitutional Genetics, Amiens University Hospital, 80000 Amiens, France; mathilde.pujalte@chu-lyon.fr (M.P.); n.celton@chu-tours.fr (N.C.); garcon.loic@chu-amiens.fr (L.G.); jedraszak.guillaume@chu-amiens.fr (G.J.); 3 Peritox UMR_I 01, CURS, Jules Verne University of Picardy, 80000 Amiens, France; 4 HEMATIM UR4666, CURS, Jules Verne University of Picardy, 80000 Amiens, France

**Keywords:** premature ovarian insufficiency, infertility, genome disorders

## Abstract

**Objective(s):** Premature ovarian insufficiency (POI), affecting 1% of women, is characterized by the loss of ovarian activity with amenorrhea or oligomenorrhea and increased gonadotropins occurring before the age of 40 years. Iatrogenic, autoimmune, and genetic causes are known to be involved in POI, but nearly 70% of all forms remain unexplained. Recent and new genetic analyses promote the identification of new candidate genes. The aim of this study was to evaluate the contribution of array-CGH and next-generation sequencing (NGS) in the diagnosis of POI. **Study design:** Twenty-eight idiopathic POI patients with primary or secondary amenorrhea underwent genetic screening by array-CGH and NGS using a custom capture design of 163 genes known or suspected to be involved in ovarian function. The clinical, biological, and ultrasound characteristics of the patients were also recorded. **Results:** Four of the twenty-eight patients had primary amenorrhea (14.3%), and twenty-four (85.7%) had secondary amenorrhea, with an average age at diagnosis of 27.7. Eleven patients (39.3%) had a family history of POI. Our study identified a genetic anomaly in 16 of 28 patients (57.1%): one patient carried a causal copy number variation (CNV), eight patients carried a causal single nucleotide variation (SNV)/indel variation (28.6%), and seven other patients carried variants of uncertain significance. **Conclusions:** Our study was the first to combine genetic analyses by using both array-CGH and NGS in the same patients. It confirmed the usefulness of both analyses in the identification of pathogenic variations responsible for idiopathic POI. Early genetic diagnosis plays a major role in the management of complications and the screening of relatives.

## 1. Introduction

Premature ovarian insufficiency (POI) is characterized by primary or secondary amenorrhea of more than 4 months before the age of 40 years associated with follicle stimulating hormone (FSH) levels greater than 25 IU/L in 2 consecutive samples [1].

Prevalence is 1–2% of women before 40 years, 0.1% before 30 years, and 0.01% before 20 years [2,3,4]. Three pathophysiological mechanisms are usually invoked: an abnormality in the constitution of the primordial follicle pool during fetal life, accelerated atresia of the follicular pool, and possible alteration in the maturation or recruitment of primordial follicles [5]. POI leads to complications such as pubertal underdevelopment, infertility, osteoporosis, and cardiovascular disease [1].

POI is a multi-factorial disease with known implications of genetic and environmental factors. In 70% of cases, the etiology has not been identified yet [6], but genetics probably plays a major role. Indeed, a familial form is identified in 12 to 31% of POI [7,8,9] and a molecular cause in 20 to 25% of POI [5,10]. Chromosomal abnormalities, including X structural anomalies and monosomy, are the most frequently identified genetic causes of POI. The second most common genetic cause is premutation of the *FMR1* gene [10]. Apart from these common causes, some syndromic POI due to pathogenic identified variants in the *NOBOX*, *BMP15*, and *GDF5* genes has been known for many years [6]. More recently, studies using array-comparative genomic hybridization (array-CGH) and next-generation sequencing (NGS) technologies have identified many new candidate genes for isolated or syndromic POI. These genes are involved in oogenesis, folliculogenesis, primordial follicle activation, meiosis, and DNA replication or repair [5,10,11,12,13,14].

The need for genetic diagnosis of POI is becoming essential to identify the etiology of POI. If a pathogenic variation is identified, familial genetic counseling and testing in relatives for the pathogenic variation are recommended [1].

The main objective of this study was to evaluate the performance of array-CGH and NGS in a panel of genes in the genetic diagnosis of POI. The secondary objective was to identify a potential association between these abnormalities and population characteristics.

## 2. Methods

We conducted an observational, retrospective single center study in the Reproductive Medicine Department of the Amiens University Hospital. The included patients presented with idiopathic POI characterized by primary amenorrhea (PA) or secondary amenorrhea (SA) of more than 4 months and FSH values > 25 IU/L before the age of 40 years. All of them had a BMI < 35. The study was approved by the local ethic committee (ID number PI2022_843_0060). Almost all patients had hormonal blood tests with FSH, 17bEstradiol, and anti-Müllerian hormone (AMH), as well as a transvaginal ultrasound with antral follicle count (AFC). Values were an average when more than one analysis was performed.

Patients with karyotype abnormalities, *FMR1* gene premutation, or iatrogenic or autoimmune causes of POI were excluded.

Genetic analyses were performed after obtaining informed consent from each patient. DNA was extracted from a peripheral blood sample using the QIAsymphony DNA midi kits on a QIAsymphony system (Qiagen^®^, Hilden, Germany). The identification of copy number variations (CNVs) was performed by oligonucleotide array-CGH using SurePrint G3 Human CGH Microarray 4 × 180 K technology following the supplier’s recommendations (Agilent Technologies, Santa Clara, CA, USA). Bioinformatics analyses were performed using Feature Extraction and CytoGenomics software v5.0 (Agilent Technologies®, Santa Clara, CA, USA) using standard settings, which allowed the detection of CNVs of a minimum of 60 kb along the genome. The identified CNVs were analyzed using the Cartagenia Bench Lab CNV software v5.1 (Agilent Technologies®).

NGS analyses to detect single nucleotide variations (SNVs) were performed using SureSelect XT-HS reagents (Agilent Technologies®) with a custom capture design of 163 genes (see list of genes in the Appendix A) on a Magnis system (Agilent Technologies) following the supplier’s recommendations. Sequencing was performed on a NextSeq 550 system (Illumina, San Diego, CA, USA). Bioinformatics analyses were performed using Alissa Align&Call v1.1 and Alissa Interpret v5.3 softwares (Alissa—Agilent Technologies®).

All identified variants were analyzed using general population databases (gnomAD, DGV), variation databases (DECIPHER, Clingen, Achropuce Network HGMD, and ClinVar), and the literature. The clinical phenotypes were compared with the data described in the Online Mendelian Inheritance in Man (OMIM) database and literature. Variants were classified according to the recommendations of the American College of Medical Genetics (class 1, benign; class 2, likely benign; class 3, variant of unknown significance (VUS); class 4, likely pathogenic; class 5, pathogenic).

This study was approved by the local ethic committee and registered with ID PI2022_843_0060.

## 3. Results

Twenty-eight women with idiopathic POI were included (Table 1). Four of them (14.3%) had PA, and 24 (85.7%) had SA, with an average age at diagnosis of 27.7 years. Eleven patients (39.3%) had a familial history of POI.

FSH levels were between 14 and 178 IU/L, with a median of 71 IU/L. Patient 6 (FSH 14 IU/L) had anorexia nervosa with functional hypothalamic amenorrhea associated with POI. AMH levels were undetectable (<0.2 ng/mL) in 78% of the 24 patients who received this analysis.

Regarding infertility and assisted reproductive technology, 23 patients (82.1%) were referred to oocyte or embryo donation. Fertility preservation by oocyte vitrification was proposed to a 24-year-old single patient, considering her preserved follicular reserve on AFC (7). Three patients did not initiate any treatment, due to their young age or personal situation. One of our patients experienced a spontaneous pregnancy.

In our study, array-CGH and NGS enabled a genetic diagnosis to be made in 16 of 28 patients (57.1%) (Table 1). The overall diagnostic yield was 75% in the PA subgroup (3/4 patients) and 45% (5/11) in patients with a familial history of POI. Patients with SA had an average age at diagnosis of 27.8 years, and a genetic abnormality was identified in 59% (13/22) of these patients.

### 3.1. Array-CGH

Array-CGH identified a CNV of interest in four patients (14.3%) (Table 1). Patient 3, who presented with PA, carried a rare 15q25.2 recurrent microdeletion, which contained the *CPEB1* gene. This CNV was classified as pathogenic and responsible for the phenotype. The other three CNVs were classified as VUSs. The 15q26.1 gain identified in patient 5 contained the *SLCO3A1* gene. The 5q13.2 multiplication identified in patient 6 included the first two exons of the *NAIP* gene. The Xp22.2 gain in patient 27 contained *FANCB* and *ASB9* genes. All of these genes had a role in ovarian function or in POI.

### 3.2. Next-Generation Sequencing

NGS revealed pathogenic or likely pathogenic variants in eight patients (28.6%) and VUSs in five other patients (17.9%). In total, a variant of interest was identified in 13 of the 28 patients, i.e., a diagnostic yield of 46.5% (Table 1).

All but one of the likely pathogenic or pathogenic variants were identified in genes known to cause POI. These genes were involved in oogenesis or folliculogenesis (*FIGLA* in patients 2 and 19 and *GALT* in patient 18) or in DNA repair and replication (*TWNK* in patient 7, *POLG* in patient 11, *ERCC6* in patient 15, and *MCM9* in patient 25). The variant in patient 22 was identified in *FOXO3*, a gene for which data on its involvement in POI are still weak. The variant was classified as probably pathogenic.

All variations classified as VUSs had characteristics that tended to classify them as pathogenic. However, they remained of uncertain significance because of an insufficient level of evidence for the genes involved or because of the presence of two to three variants in distinct genes in the same patient.

## 4. Discussion

Our cohort included 28 patients with idiopathic POI selected according to strict pre-defined clinical criteria. To our knowledge, this study was the first to combine genetic analyses by array-CGH and NGS using a panel of genes previously implicated in POI and/or ovarian function in patients with POI. This combination of analyses enabled a genetic diagnosis to be made in 57.1% of the cases.

In our cohort, 4 patients (14.3%) had PA, and 24 (85.7%) had SA. In the SA subgroup, the average age at diagnosis was 27.7 years. We did not include women suffering from diminished ovarian reserve (DOR) without amenorrhea in order to select the most severe phenotypes and increase the diagnostic yield. Among other studies including patients with primary and secondary amenorrhea, the percentage of PA varied between 6% and 58%, while the mean age at diagnosis of SA was between 22 and 29 years [15,16,17,18,19]. In our study, 39% of patients had a family history of POI. This was a high rate compared with other studies. Indeed, other studies showed great variability, with rates ranging from 6 to 37% [15,16,20,21,22,23].

A retrospective study conducted in our center on 432 patients with POI showed that karyotype, FISH, and *FMR1* premutation research identified a cause of POI in only 13% of cases, which corresponded to results previously reported in the literature [10,24] In the present study, 16 of the 28 studied patients who had negative results for common genetic explorations carried at least one variant of interest (57.1%).

Array-CGH identified a CNV of interest in four patients (14.3%). One of these variants was considered pathogenic (patient 3), and three were classified as VUSs. The pathogenic CNV was a 15q25.2 microdeletion including the *CPEB1* gene, which is known to be involved in female meiosis and follicular atresia [21,25,26]. This recurrent microdeletion has been reported in several cases in patients with POI [21,25,27,28]. Our result reinforced the pathogenicity of this deletion and showed the importance of looking for it in patients with POI, especially in cases of primary or early amenorrhea. Three CNVs classified as VUSs were identified in our patients: 15q26.1 gain including *SLCO3A1*, multiplication of 5q13.2 including *NAIP*, and Xp22.2 gain including *FANC* and *ASB9* genes. These CNVs are non-recurrent chromosomal variants containing candidate genes for POI. *SLCO3A1* is involved in the transport of prostaglandins E1 and E2, and a deficiency could be the cause of impaired folliculogenesis. However, no mutation, deletion, or duplication of this gene has been described in human disease. A study has already identified the multiplication of the *NAIP* locus in two patients with POI [15]. One hypothesis is that *NAIP* plays a role in oocyte survival by inhibiting granulosa cell apoptosis during folliculogenesis [29]. *FANCB* encodes a protein involved in DNA repair, and other genes in the FANC family have already been implicated in POI [30]. *ASB9* encodes a protein that is a negative regulator of ovarian granulosa cell proliferation and differentiation, and its deregulation could impact luteal cell differentiation [31,32,33]. For all these VUSs, further studies are needed to form conclusions regarding their involvement in POI in our patients. The overall yield of array-CGH in our study was 14.3% and within the range of known yields in the literature. In the eight studies of POI patients explored by array-CGH, CNV identification varied from 5.6 to 59% of cases [15,16,20,21,22,27,28,34] (Table 2). This variability can be explained by the patient selection criteria, the type of array used, and the criteria for CNV classification (inclusion or exclusion of VUSs). Our results were comparable to those of the Jaillard et al. study, which was carried out on 60 infertile women requiring oocyte donation because of reduced ovarian reserve. The same array-CGH technology was used [20]. They identified CNVs of interest in 15% of the cases.

In our study, we analyzed a panel of 163 genes involved in POI. We identified a variant of interest in 13 patients (46.5% of the cohort). In eight patients, a likely pathogenic variant (class 4) or a pathogenic variant (class 5) was identified. In addition, at least one VUS (class 3) was identified in five other patients. All but one pathogenic or likely pathogenic variant were identified in genes known to be involved in POI (*FIGLA*, *GALT*, *TWNK*, *POLG*, *ERCC6*, and *MCM9*). The only likely pathogenic variant identified, in a gene whose implication in POI is still debated, was the variant in *FOXO3* in patient 22. This variant was classified as likely pathogenic because of its description in a previous study and due to its proven functional impact [35]. Only two of these variants have been reported in the literature to date. One patient (patient 5) had two previously unreported VUSs in the heterozygous state in two genes, *PMM2* and *DMC1*, known to cause autosomal recessive POI. We also identified variants of interest in genes whose relationship with POI was described but still uncertain: *MACF* [20], *NBN* [36], *EIF2B2* [37], *RECQL4* [20], *TUBB8* [38], *SALL4* [39], *EIF4ENIF1* [40], and *ATM* [41]. We considered these genes to be uncertainly responsible for POI as they have been reported only in few cases and/or a few studies on large cohorts that identify variations of interest of these genes.

In the literature, eight studies on a cohort of women with POI have investigated the frequency of pathogenic gene variants. The diagnostic yield is between 19 and 70% of cases [17,18,19,23,41,42,43,44] (Table 3). These variable results are due to selection of patients (inclusion criteria) and variable analyses (18-gene panel to whole exome), which makes comparisons difficult. Nevertheless, the 46.5% yield in our study was comparable to those of other studies using similar panel sizes. Analyses of large gene panels show significant genetic heterogeneity. Some genes, such as *BMP15*, *NOBOX*, *FSHR*, *GDF9*, *MCM8*, *MCM9*, *STAG3*, or *HFM1* [10,13,14,18], were found in each study, which underlined their importance in genetic causes of POI. Other genes were less frequently implicated, and the evidence for causality remains to be demonstrated. Our study, with the identification of variants in *MACF1*, *RECQL4*, *TUBB8*, *SALL4*, *ATM*, or *FOXO3*, reinforced the hypothesis that these genes were involved in POI. Our study highlighted the use of a combination of new genetic technologies added to conventional explorations, leading to a considerable increase in the diagnostic yield in POI, with the identification of a potential cause in nearly 57.1% of cases. This yield could increase with the identification of new candidate genes for future studies on the subject, which will require constant adaptation of the studied gene panels. In addition, new technologies such as whole genome sequencing (WGS) or optical genome mapping will probably enable the identification of variants not currently identified by karyotype, CGH, or WES (small balanced chromosomal variants, intronic, or intergenic variants). Furthermore, it seems to be necessary to plan analyses in the future that can (i) confirm the functional impacts (cellular assays and animal studies) of all variations identified in genes panels, particularly for VUSs, and (ii) use all new technologies available for large genomic exploration such as multi-omic analyses.

Expectedly, the genetic diagnosis of POI was more frequent in PA patients. Our diagnostic yield in this subgroup was 25% in array-CGH, 50% in NGS, and 75% in total. These results must be moderated because of the very small number of patients. However, a large study also found a higher rate of genetic forms in patients with PA (30%) than in the general population (19%) [17]. In our study, the mean age of SA in patients with variation (27.8 years) was similar to that of SA in the entire cohort (27.7 years). Moreover, genetic forms of POI were not more frequent in cases of family history (overall diagnostic yield of 45% in the 11 patients reporting a family history versus 53.6% for the entire cohort). Eskenasi’s study, which included a population of 269 patients, showed no significant difference in the genotype/phenotype correlation in mutations [23] All these elements confirm the complexity of the mechanisms of POI.

Our study focused on the identification of single variants responsible for POI. Nevertheless, it is widely accepted that these genetic causes have incomplete penetrance and variable expressivity, even within the same family. There are several hypotheses to explain these phenomena, and one is the possibility of an oligogenic or polygenic origin of POI. Of our population, 21.4% (6/28) had more than one variant of interest. One of our patients carried three VUSs, one identified by array-CGH and two identified by NGS. Other studies have already reported patients carrying more than one variant [14,17,21,43]. However, patients carrying several mutations did not have more severe phenotypes (all six in SA, mean age at SA consisted of 30.16% familial forms). Contradictory data are reported in the literature on this subject. The Eskenasi study did not show a dose effect in cases of multigenism, while the Bouilly study found a higher proportion of PA and a younger mean age at SA diagnosis in patients carrying at least two variants [17,23]. These results highlight the hypothesis of a potentially cumulative effect of rare variants in ovarian expressed genes on the occurrence of POI. Furthermore, it is possible that environmental and/or epigenetic factors add to a predisposition provided by a pathogenic variant with incomplete penetrance. Transdisciplinary studies of large cohorts of patients will be important to validate these hypotheses.

One of the major limitations of our study was the small size of the cohort. We studied patients with a definite and marked phenotype, which reduced the number of included patients. As a consequence, we believe that these results give us a better idea of the genetic yield in this population. However, these results need to be confirmed on a larger cohort.

## 5. Conclusions

In cases of idiopathic POI, array-CGH and NGS identify an etiology in 5 to 70% of cases. Our study showed a contribution of these new diagnostic methods in 57.1% of our cases. While 70% of POI are still defined as idiopathic, these results appear encouraging. In POI patients, genetic screening combining array-CGH and NGS could be considered as a second-line etiological assessment. It could be used in non-syndromic forms with familial counselling in the case of a positive diagnosis. These methods could also be extended to patients with DOR.

The genetic diagnosis of POI is an important issue in the management of this disorder. In these patients who are often suffering from wandering diagnostics and difficult experiences, acceptance of the disease could be facilitated by an etiological diagnosis. Moreover, the early genetic diagnosis of POI and screening of relatives makes sense, considering the rise of fertility preservation methods, notably oocyte vitrification.

Some of the genes in our panel were involved in both the mechanisms of POI and cancer predisposition through their roles in meiosis and DNA repair. Thus, the discovery of a variant in one of these genes could justify appropriate screening measures to limit the oncological risk in patients and their family.

## Figures and Tables

**Table 1:** Population characteristics and genetic results. FSH, follicle-stimulating hormone; AMH, anti-Müllerian hormone; NGS, next-generation sequencing; PA, primary amenorrhea; SA, secondary amenorrhea; VOI, variation of interest; NA, not available. Genetics data are presented using the GRCh37/hg19 genome version.

Patient	Amenorrhea	Age at Diagnosis	FSH IU/L	17bE2 pg/mL	AMH ng/mL	Familial History	Array-CGH		NGS	
							Variation	Classification	Variation	Classification
1	SA	28	34	18	0	NA	No VOI		No VOI	
2	PA	NA	58	7	<0.1	+	No VOI		Homozygous *FIGLA* variation Chr2(GRCh37):g.71014926dup NM_001004311.3(FIGLA):c.239dup p.(Asn80Lysfs*26)	Pathogenic Class 5
3	PA	NA	178	14	0	NA	15q25.2 deletion arr[GRCh37] 15q25.2(83240239_85090038)x1	Pathogenic Class 5	No VOI	
4	SA	15	25	11	<0.1	+	No VOI		No VOI	
5	SA	25	72	29	0.1	-	15q26.1 gain arr[GRCh37] 15q26.1(92521854_92810021)x3	VUS Class 3	Heterozygous *PMM2* variation Chr16(GRCh37):g.8895680T>C NM_000303.3(PMM2):c.91T>C p.(Phe31Leu)	VUS Class 3
									Heterozygous *DMC1* variation Chr22(GRCh37):g.38945934T>C NM_007068.4(DMC1):c.490A>G p.(Thr164Ala)	VUS Class 3
6	SA	32	14	1	<0.1	-	5q13.2 gain arr[GRCh37] 5q13.2(70309796_70657747)x4	VUS Class 3	No VOI	
7	SA	30	166	0	NA	+	No VOI		Heterozygous *TWNK* variation Chr10(GRCh37):g.102749177G>C NM_001163812.2(TWNK):c.1210G>C p.(Gly404Arg)	Likely Pathogenic Class 4
8	SA	13	141	0	NA	-	No VOI		No VOI	
9	SA	32	58	26	0.7	-	No VOI		Heterozygous *MACF1* variation Chr1(GRCh37):g.39889857C>T NM_012090.5(MACF1):c.10121C>T p.(Ala3374Val)	VUS Class 3
									Heterozygous *NBN* variation Chr8(GRCh37):g.90990521T>C NM_001024688.3(NBN):c.265A>G p.(Ile89Val)	VUS Class 3
10	SA	28	76	18	0.2	-	No VOI		No VOI	
11	SA	23	84	37		-	No VOI		Heterozygous *POLG* variation Chr15(GRCh37):g.89873337T>A NM_002693.3(POLG):c.830A>T p.(His277Leu)	Likely Pathogenic Class 4
12	SA	24	119	6	<0.2	-	No VOI		No VOI	
13	SA	26	NA	NA	<0.1	-	No VOI		Heterozygous *EIF2B2* variation Chr14(GRCh37):g.75475866A>G NM_014239.4(EIF2B2):c.1031A>G p.(Tyr344Cys)	VUS Class 3
									Heterozygous *RECQL4* variation Chr8(GRCh37):g.145742547G>A NM_004260.4(RECQL4):c.241C>T p.(His81Tyr)	VUS Class 3
14	SA	34	25	14	0.48	+	No VOI		Heterozygous *TUBB8* variation Chr10(GRCh37):g.93488G>A NM_177987.3(TUBB8):c.844C>T p.(Arg282Trp)	VUS Class 3
									Heterozygous *SALL4* variation Chr20(GRCh37):g.93488G>A NM_020436.5(SALL4):c.2677_2678delAC p.(Thr893Trpfs*32)	VUS Class 3
15	SA	30	129	0	0	-	No VOI		Heterozygous *ERCC6* variation Chr10(GRCh37):g.50724287G>A NM_001277058.2(ERCC6):c.2278C>T p.(Arg760Trp)	Likely Pathogenic Class 4
16	SA	36	32	48	<0.1	+	No VOI		No VOI	
17	SA	33	120	15	<0.1	-	No VOI		Heterozygous *ATM* variation	VUS Class 3
									Heterozygous *EIF4ENIFI* variation	VUS Class 3
18	SA	35	111	24	<0.1	-	No VOI		Heterozygous *GALT* variation Chr9(GRCh37):g.34647855C>T NM_000155.4(GALT):c.404C>T p.(Ser135Leu)	Pathogenic Class 5
									Heterozygous *ATM* variation Chr11(GRCh37):g.108202207C>T NM_001351834.2(ATM):c.7552C>T p.(Pro2518Ser)	VUS Class 3
19	PA	NA	41	0	0.3	+	No VOI		Homozygous *FIGLA* variation Chr2(GRCh37):g.71014926dup NM_001004311.3(FIGLA):c.239dup p.(Asn80Lysfs*26)	Pathogenic Class 5
20	SA	30	76	0	<0.1	+	No VOI		No VOI	
21	SA	31	178	14	<0.1	+	No VOI		No VOI	
22	SA	18	153	0	0	-	No VOI		Heterozygous *FOXO3* variation Chr6(GRCh37):g.108985553G>A NM_201559.3(FOXO3):c.1517G>A p.(Arg506His)	Likely Pathogenic Class 4
23	PA	NA	107	0	NA	+	No VOI		No VOI	
24	SA	35	107	0	<0.1	-	No VOI		No VOI	
25	SA	16	70	10	<0.1	+	No VOI		Heterozygous *MCM9* variation Chr6(GRCh37):g.119243238G>A NM_001378357.1(MCM9):c.635C>T p.(Ser212Phe)	Likely Pathogenic Class 4
26	SA	35	114	3	0	-	No VOI		No VOI	
27	SA	24	25	37	0.6	-	Xp22.2 gain arr[GRCh37] Xp22.2(14652955_15279642)x3	VUS Class 3	No VOI	
28	SA	33	96	13	<0.1	+	No VOI		No VOI	

**Table 2:** Main studies investigating the contribution of array-CGH in premature ovarian insufficiency. PA, primary amenorrhea; SA, secondary amenorrhea; CNV, copy number variations; NA, not available.

	Patients (n)	Amenorrhea	Mean Age at SA	CNV Yield (%)
Aboura et al. 2009 [15]	99	PA and SA	25	8
Ledig et al. 2010 [22]	74	NA	NA	59
Liao et al. 2011 [34]	15	PA	NA	26
McGuire et al. 2011 [27]	89	PA and SA	NA	27
Norling et al. 2014 [16]	26	PA and SA	22	42
Tsuiko et al. 2016 [28]	301	SA	37	5.6
Jaillard et al. 2016 [20]	60	PA and SA, oligomenorrhea	30	15
Bestetti et al. 2019 [21]	67	PA	16	47.8

**Table 3:** Main studies investigating the contribution of next-generation sequencing in premature ovarian insufficiency. PA, primary amenorrhea; SA, secondary amenorrhea; WES, whole exome sequencing; NA, not available.

	Patients (n)	Amenorrhea	Mean Age at SA	Number of Sequencing Genes	Yield of Variant of Interest (%)
Fonseca et al. 2015 [42]	12	NA	NA	70	33.3
Bouilly et al. 2016 [17]	100	PA and SA	29	19	19
Patino et al. 2017 [43]	69	PA and SA	NA	420	48
Jolly et al. 2019 [44]	42	NA	NA	WES	13
Liu et al. 2020 [41]	24	PA and SA	NA	WES	58.3
França et al. 2020 [18]	50	PA and SA	27	165	70
Shen et al. 2021 [19]	73	PA and SA	25	60	27.4
Eskenazi et al. 2021 [23]	269	PA and SA	NA	18	38

## Data Availability

The original contributions presented in the study are included in the article, further inquiries can be directed to the corresponding author.

## Appendix A. List of Genes Included in the Panel

*AARS2*	*POLG*	*DACH2*	*GNRHR*	*NOG*	*SOX10*
*BMP15*	*SOHLH1*	*DCAF17*	*GPR3*	*NOTCH2*	*SOX2*
*BNC1*	*STAG3*	*DMC1*	*HAMP*	*NR0B1*	*SOX3*
*CLPP*	*TWNK*	*DMXL2*	*HESX1*	*NRIP1*	*SPANXC*
*CPEB1*	*DIAPH2*	*DUSP6*	*HFE*	*NSMF*	*SPIDR*
*CYP17A1*	*ADAMTSL1*	*EIF2B2*	*HFE2*	*NUP107*	*SPO11*
*CYP19A1*	*AFF2*	*EIF2B3*	*HS6ST1*	*PGRMC1*	*SPRY4*
*EIF2B5*	*AIRE*	*EIF2B4*	*IL17RD*	*POLR2C*	*STAR*
*ERCC6*	*AMH*	*EIF4ENIF1*	*INHA*	*POLR3H*	*SYCE1*
*FANCM*	*AMHR2*	*ERAL1*	*KHDRBS1*	*POU5F1*	*TAC3*
*FIGLA*	*ANOS1*	*ESR1*	*KIAA0391*	*PREPL*	*TACR3*
*FOXL2*	*ANTXR1*	*FANCA*	*KISS1*	*PROK2*	*TFR2*
*FSHB*	*APBA3*	*FANCC*	*KISS1R*	*PROP1*	*TG*
*FSHR*	*AR*	*FANCG*	*KITLG*	*PSMC3IP*	*TGFBR3*
*GALT*	*ATG7*	*FER1L6*	*KLB*	*PTRD*	*TP63*
*GDF9*	*ATG9A*	*FEZF1*	*LHB*	*RBBP8*	*TUBB8*
*HARS2*	*ATM*	*FGF17*	*LHCGR*	*RCBTB1*	*WDR11*
*HFM1*	*AXL*	*FGF8*	*LHX3*	*REC8*	*WDR62*
*HSD17B4*	*BLM*	*FGFR1*	*LHX4*	*RECQL4*	*WNT4*
*LARS2*	*BMPR1B*	*FLRT3*	*LHX8*	*SALL4*	*WRN*
*MCM8*	*BMPR2*	*FMR1*	*LMNA*	*SEMA3A*	*WT1*
*MCM9*	*BUB1B*	*FOXO1*	*MACF1*	*SEMA3E*	*XPO1*
*MLH3*	*CCDC141*	*FOXO3*	*MRPS22*	*SEMA7A*	*XRCC4*
*MSH4*	*CDKN1B*	*FOXO4*	*MSH5*	*SGO2*	
*NOBOX*	*CHD7*	*GJA4*	*NAIP*	*SLC29A3*	
*NR5A1*	*CITED2*	*GLI2*	*NANOS3*	*SLC40A1*	
*PMM2*	*CLDN14*	*GNAS*	*NBN*	*SMC1B*	
*POF1B*	*CUL4B*	*GNRH1*	*NDNF*	*SOHLH2*	
